# A Protocol Investigation Comparing Transcatheter Repair with the Standard Surgical Procedure for Secondary Mitral Regurgitation

**DOI:** 10.3390/jcm13247742

**Published:** 2024-12-18

**Authors:** Francesco Nappi, Sanjeet Singh Avtaar Singh, Antonio Salsano, Aubin Nassif, Yasushige Shingu, Satoru Wakasa, Antonio Fiore, Cristiano Spadaccio, Zein EL-Dean

**Affiliations:** 1 Department of Cardiac Surgery, Centre Cardiologique du Nord., 93200 Saint-Denis, France; aubinnassif@gmail.com; 2 Department of Cardiothoracic Surgery, Royal Infirmary of Edinburgh, Edinburgh EH16 4SA, UK; sanjeetsinghtoor@gmail.com; 3 Division of Cardiac Surgery, DISC Department, University of Genoa, 16132 Genova, Italy; ant.salsano@gmail.com; 4 Department of Cardiovascular Surgery, Faculty of Medicine and Graduate School of Medicine, Hokkaido University, Sapporo 060-8638, Japan; yshingu@ac.cyberhome.ne.jp (Y.S.); wakasa@med.hokudai.ac.jp (S.W.); 5 Department of Cardiac Surgery, Hopital Henri Mondor, Assistance Publique—Hopitaux de Paris, 94000 Créteil, France; antonio.fiore@aphp.fr; 6 University of Cincinnati Medical Center, 2600 Clifton Ave, Cincinnati, OH 45221, USA; spadacco@ucmail.uc.edu; 7 Department of Cardiothoracic Surgery, Mayo Clinic, 200 1st St SW, Rochester, MN 55905, USA; 8 Department of Cardiac Surgery, Glenfield Hospital, University Hospitals of Leicester, Leicester LE3 9QP, UK; zein.eldean@hotmail.co.uk

**Keywords:** secondary mitral regurgitation, restrictive mitral annuloplasty, subvalvular repair, mitral valve replacement, transcatheter edge-to-edge repair

## Abstract

**Background:** Secondary mitral regurgitation (SMR) is characterized by a pathological process impacting the left ventricle (LV) as opposed to the mitral valve (MV). In the absence of structural alterations to the MV, the expansion of the LV or impairment of the papillary muscles (PMs) may ensue. A number of technical procedures are accessible for the purpose of determining the optimal resolution for MR. Nevertheless, there is a dearth of rigorous data to facilitate a comparative analysis of MV replacement, MV repair (including subvalvular repair), and transcatheter mitral valve interventions (TMV-Is). The objective of this investigation is to evaluate and compare the efficacy and clinical outcomes of transcatheter mitral valve repair (TMV-r) utilizing the edge-to-edge mitral valve repair (TEER) procedure in comparison to conventional surgical mitral valve interventions (S-SMVis) in patients with secondary mitral regurgitation. **Methods and analysis:** A consortium of five cardiac surgery institutions from four European states and Japan have joined forces to establish a multicenter observational registry, designated TEERMISO. Patients who underwent technical procedures for SMR between January 2007 and December 2023 will be enrolled consecutively into the TEERMISO registry. The investigation team evaluated the comparative efficacy of replacement and repair techniques, utilizing both the standard surgical methodology and the transcatheter intervention. The primary clinical outcome will be the degree of left ventricular remodeling, as assessed by the left ventricular end-diastolic volume index, at 10 years. The forthcoming research will assess a variety of secondary endpoints, among which all-cause mortality will be the primary endpoint. Subsequent assessments will be made in the following order: functional status, hospitalization, neurocognition, physiological measures (echocardiographic assessment), occurrence of adverse clinical incidents, and reoperation. **Ethics and dissemination:** The multicenter design of the database is anticipated to reduce the potential for bias associated with institutional caseload and surgical experience. All participating centers possess an established mitral valve protocol that facilitates comprehensive follow-up and management of any delayed mitral complications following replacement surgery or surgical repair of the secondary mitral regurgitation. The data collected will provide insights into the impact of diverse surgical approaches on standard mitral valve surgery and TEER. This will facilitate the evaluation of LV remodeling over the course of long-term post-procedural follow-up. **Trial Registration:** ClinicalTrials.gov ID: NCT05090540; IRB ID: 202201143

What is already known on this topic? The debate over therapeutic interventions to correct secondary mitral regurgitation continues. Currently, the guidelines lack reliable long-term follow-up data. This study aims to compare the long-term clinical outcomes of patients who underwent transcatheter mitral valve procedures with those who underwent standard surgical mitral valve interventions.

What this study adds. This study will determine which of the two procedures achieves an immediate reduction in mitral regurgitation to moderate or lower levels and what percentage of patients can benefit from this result.

How this study might affect research, practice, or policy. This study will provide key information on the best treatment option between conventional mitral valve procedures and transcatheter mitral valve repair. The results can provide valuable data for the development of international guidelines.

## 1. Introduction

Secondary mitral regurgitation (SMR) is a condition that arises when the left ventricle (LV) undergoes enlargement or when the papillary muscles are compromised without any structural alterations to the mitral valve (MV). The principal characteristic of this condition is a significant alteration in the spatial arrangement of the constituent elements of the MV and subvalvular (SV) apparatus. The primary treatments for heart failure (HF) patients with SMR are guideline-directed medical therapy (GDMT), cardiac resynchronization, and coronary revascularization, which are used to optimize the treatment of the underlying LV dysfunction. The question of whether therapeutic measures aimed at improving SMR have an impact on subsequent clinical outcomes remains a topic of debate due to the fact that the disease mechanism is limited to the LV as opposed to the MV [1,2,3].

The initial and indispensable phase in the administration of all patients with SMR is the implementation of optimized pharmacological regimens in accordance with the established treatment guidelines for heart failure. This process should entail the substitution of an ACEI or ARB with sacubitril/valsartan, sodium–glucose co-transporter 2 inhibitors, and/or ivabradine if indicated [2,3].

There is no definitive evidence to support surgery for the treatment of SMR. Although restrictive mitral annuloplasty (RMA) is the most commonly used conservative technique for the standard mitral valve intervention (S-MVi), observational studies have shown that it is effective in reducing SMR and improving symptoms. However, it is uncertain whether these results are sustainable or whether the technique improves mortality compared with GDMT [4,5,6,7].

Further investigation of the long-term effects of RMA on patient outcomes is necessary to provide robust support for its use, given the conflicting results that currently exist. Patients with atrial functional mitral regurgitation typically present with a normal LVEF, less pronounced LV dilatation, and mitral annular dilatation as the primary mechanism of mitral regurgitation. This subgroup may benefit from RMA, which is frequently combined with the ablation of AF, although evidence remains limited [2]. A total of 251 patients were enrolled in the CTS Network randomized clinical trial (RCT) across 23 countries [8]. The principal objective was to ascertain the extent of reverse remodeling of the LV, as indicated by LVESV 12 months after randomization. At the two-year interim analysis, no significant intergroup difference in left ventricular reverse remodeling was observed. This was evidenced by a mean change of −6.5 mL and −9.0 mL from baseline in the chordal-sparing mitral valve repair (CS-MVRpl) and surgical mitral valve (SMV) repair (S-MVr) groups, respectively [8]. The results of the CTSN trial beyond the two-year mark will not be published due to the limitations of the study design, which did not allow for a follow-up period exceeding two years. Chordal-sparing mitral valve replacement may prove an advantageous alternative to downsizing annuloplasty repairs in patients diagnosed with coronary artery disease (CAD) and chronic severe SMR due to LV systolic dysfunction < 50% (Stage D) who require mitral valve surgery due to severe clinical symptoms (New York Heart Association [NYHA] class III or IV) that fail to remit despite optimal medical therapy for heart failure [8]. In accordance with the American College of Cardiology/American Heart Association (ACCA) guidelines, the recommendation for surgical valve replacement is classified as class of recommendation (COR) 2b and level of evidence (LOE) B-R [2,3] (Table 1).

In addition, there is a range of surgical procedures that can be employed in the treatment of mitral valve disease [9,10,11,12,13]. An RCT revealed that S-MVr was linked to comparable long-term outcomes in terms of survival rates. Nevertheless, the study also indicated an elevated likelihood of recurrent MR and cardiovascular rehospitalization in patients who underwent S-MVr in comparison to those who received chordal-sparing mitral valve replacement [8]. Nevertheless, the selection of the appropriate surgical technique remains contingent upon the experience and expertise of the surgical center and the attending surgeon [1,2,3]. In view of the paucity of clinical evidence attesting to the efficacy of two-stage MV repair, which entails a combination of subvalvular repair (SV-r) and RMA, surgeons may be disinclined to adopt this technique as a means of addressing MV issues. This conclusion is supported by observational studies [9,10] and a limited series of RCTs [11,12,13].

Two recently conducted RCTs have been completed: the Multicenter Study of Percutaneous Mitral Valve Repair MitraClip Device in Patients With Severe Secondary Mitral Regurgitation (MITRA-FR) and the Cardiovascular Outcomes Assessment of the MitraClip Percutaneous Therapy for Heart Failure Patients With Functional Mitral Regurgitation (COAPT). The efficacy of the transcatheter mitral valve (TMV) intervention (TMV-i) utilizing the MitraClip system (Abbott, IL, USA) was assessed in conjunction with GDMT and contrasted with GDMT alone [14,15,16,17]. The addition of percutaneous repair to medical therapeutic regimens did not result in a statistically significant reduction in the risk of death or heart failure readmission compared with medical treatment administered alone at the one- and two-year follow-ups in the MITRA-FR RCT [14,15]. Nevertheless, the COAPT RCT demonstrated that TEER was a safe intervention for patients with HF and moderate-to-severe or severe SMR who had not responded to GDMT. The intervention resulted in a reduced incidence of hospitalization for HF and all-cause mortality at 2 [16] and 5 years of follow-up compared to medical therapeutic regimens alone [17]. The COAPT trial represents the inaugural RCT to indicate a survival advantage resulting from the correction of secondary mitral regurgitation by means of transcatheter edge-to-edge repair. It is crucial to emphasize that the novel conceptualization of SMR pathology proposed by Grayburn et al. [18], in relation to the discrepancy in outcomes observed in the two RCTs, has prompted a renewed focus on the “MV-LV unit” and their functional interrelationships. It is of the utmost importance to contemplate these elements, as they introduce the notion of proportionate or disproportionate mitral regurgitation in the context of left ventricular size [18,19,20]. This concept serves to elucidate the prognosis of SMR, as well as to explain the differing results observed in the MITRA-FR [14,15] and COAPT [16,17] studies (Table 2).

A lack of available data precludes a direct comparison between TEER and traditional surgical techniques for the treatment of SMR. This encompasses the surgical intervention designed to address papillary muscle dislocation [7,9,10,11,12,13] and a subgroup analysis of patients who underwent CABG in comparison to those who did not [6]. Additionally, a notable proportion of patients who were enrolled in RCTs to receive TEER did not undergo PCI [14,15,16,17]. To date, only one study employed propensity score matching, which encompassed the surgical procedure of RMA [21]. In contrast, the other study did not utilize propensity score matching and centered on the edge-to-edge technique [22] and MV replacement [23].

It is hypothesized that the utilization of two distinct methodologies, based on either TEER or S-MVi, may result in disparate outcomes pertaining to survival rates, functional outcomes, and results obtained through echocardiography in the context of SMR treatment. The multicenter registry of transcatheter versus standard surgical mitral valve operation for secondary mitral regurgitation (TEERMISO) has been designed to assess the clinical outcomes of patients who received TEER using the MitraClip system in comparison to those receiving surgical mitral valve repair or replacement through a range of techniques for the treatment of secondary mitral regurgitation. This investigation presents a comparative analysis of the long-term clinical outcomes of two propensity score-matched cohorts, employing LASSO regression on a robustly sampled population cohort.

## 2. Methods and Analysis

The TERMISIO prospective observational registry encompasses individuals who have been subjected to technical procedures and treatments for SMR at five cardiac surgery centers located in two European nations (France and Italy) and Japan (Table 3) over the course of nine years, from 1 January 2014 to 31 March 2023. The objective of this study is to collate data on patients who had undergone treatment during the specified period.

The goal is to obtain additional data for the purpose of future clinical research on the topic in question. The data pertaining to the successive patients with SMR will be compiled in an Access database created using Microsoft Access software, developed by Microsoft in its Redmond, Washington, USA, headquarters. The data sheet will comprise a set of predetermined variables, including those pertaining to the baseline, surgical procedure, and postoperative course of the patients. Prior to the commencement of this study, permission will be sought from the relevant institutional review board or local ethical committee in accordance with the legislation of the country in which this study is to be conducted.

### 2.1. TEERMISO Study Patient Entry Criteria

#### 2.1.1. Describing Patient Groups

The clinical trial will include patients presenting with moderate to severe ischemic mitral regurgitation who may or may not require concomitant coronary artery bypass surgery (CABG) or percutaneous coronary intervention (PCI). Furthermore, patients will have undergone either S-SVRp or TMVp using TEER. Patients of any gender, race, or ethnicity will be eligible for inclusion in this study.

#### 2.1.2. Inclusion Criteria

- Age 18 years or older.
- Chronic severe ischemic and non-ischemic mitral regurgitation (EROA ≥ 0.4 cm echocardiogram) with tethering as a major mechanism.
- The occurrence of symptoms that indicate secondary mitral regurgitation (3+ or 4+ as determined by echocardiographic laboratory assessment) as an outcome of the development of cardiomyopathy, whether of an ischemic or non-ischemic etiology.
- The management of the subject was conducted in accordance with the relevant clinical guidelines, including those pertinent to CAD, LV functional impairment, MR, and HF.
- The procedure was conducted using either a standard surgical approach or a transcatheter technique for mitral valve repair.
- Patients with atrial functional mitral regurgitation.
- CAD in patients who underwent an S-MVi, with or without the need for coronary revascularization.
- It will fall upon the cardiac team at each center to ascertain which patients to include in the register. The choice of treatment will be dependent on whether the patient received a standard surgical approach or a transcatheter technique for mitral valve repair.
- In order to be included in the TEERMISO registry, it will be necessary for the individual in question to possess the capacity to sign both the informed consent and the Medical Information Release forms.

#### 2.1.3. Exclusion Criteria

- Pediatric.
- Any echocardiographic evidence of structural (chordal or leaflet) mitral valve disease.
- Papillary muscle rupture.
- Infective endocarditis.

A review of the clinical facilities participating in this investigation suggests that the aggregate sample size will be 630. The patient cohort was derived from the databases of the five participating centers, which were subjected to a rigorous screening process to ascertain their eligibility for integration into the analytical framework (Supplementary Material). A combination of monitoring strategies will be employed to oversee the patients, which may include the dissemination of information by mail to the referring physicians of patients who underwent a procedure at the hospitals, direct observation at the center of the surgical procedure, and follow-up telephone interviews with the healthcare providers where the patients were readmitted due to post-procedural complications. The research team conducted periodic assessments of the actual inclusions in comparison to the pre-specified targets. The implementation of a uniform protocol across all centers enabled the documentation of the number of patients who underwent a range of interventions and who were subjected to echocardiographic and clinical follow-up.

The repository of data encompasses information pertaining to the participation of females and ethnic minorities in scientific investigations, specifically clinical trials. This is important for a number of reasons, including those of a scientific, ethical, and social nature, as well as with a view to ensuring the generalizability of the results of the studies conducted. The TEERMISO consortium is dedicated to achieving scientific objectives while ensuring an equitable recruitment of patients, regardless of gender or ethnicity. A minimum of 30% of the TEERMISO registry participants are women, and 25% are from minority ethnic groups. In order to ensure the appropriate representation of these groups, the recruitment centers must implement two measures. Firstly, the number of females and ethnic minorities chosen and enrolled through the screening and exclusion procedures must be recorded. Secondly, these figures must be overseen by each clinical facility on an annual basis. The criteria for inclusion and exclusion of patients who have undergone technical procedures for secondary mitral regurgitation are outlined in the following section (Table 4).

### 2.2. Trial Design and Endpoints

**Figure 1:**
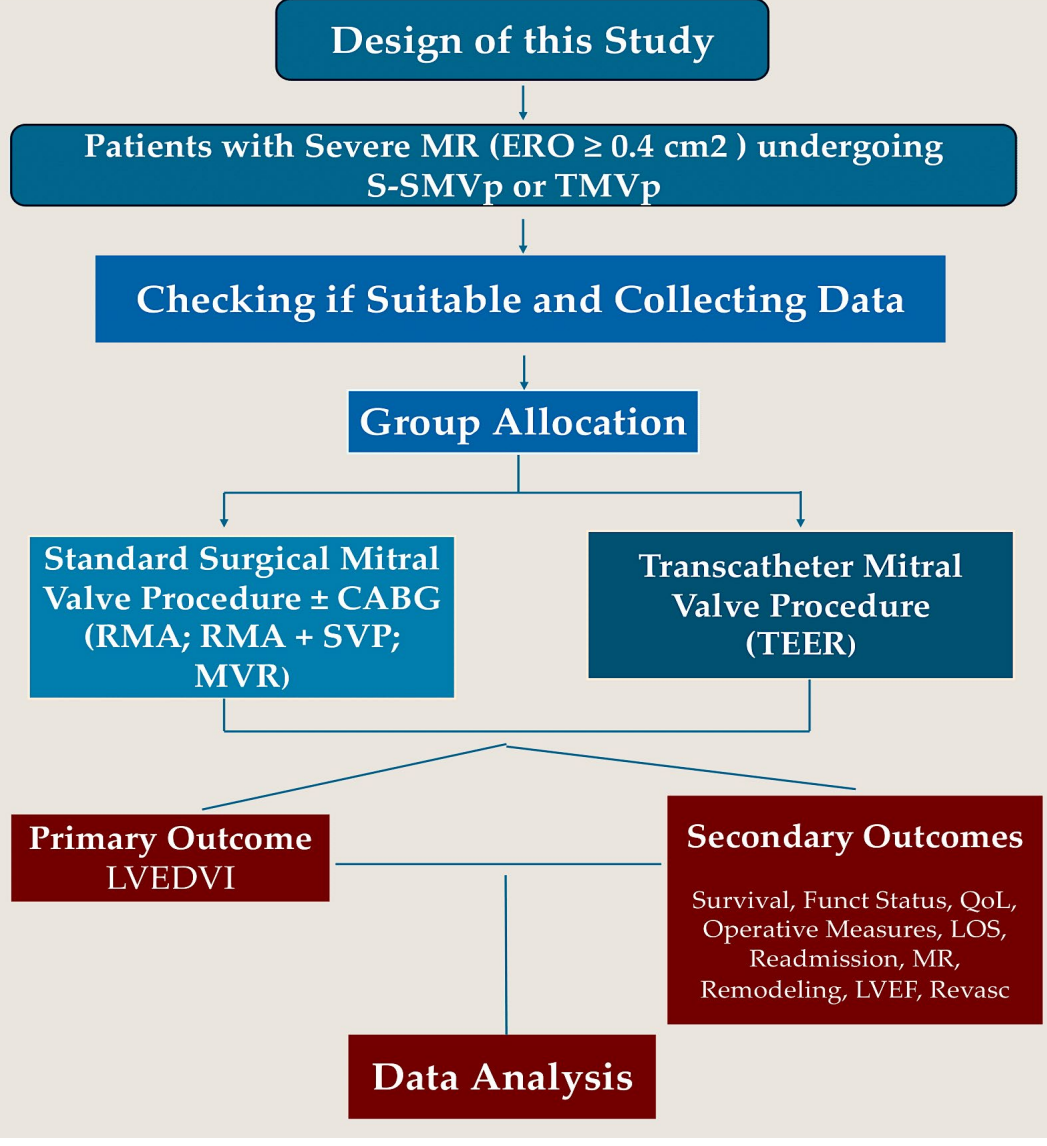
Study design schematic.

A schematic representation of the trial design is presented in Figure 1.

#### 2.2.1. Primary

The primary endpoint of this study is the degree of LV remodeling as assessed by the Left Ventricular End-Diastolic Volume Index (LVEDVI) at the 10-year follow-up.

#### 2.2.2. Secondary

A number of secondary endpoints will be subjected to evaluation in the context of the forthcoming trial. The trial will evaluate the primary secondary endpoint, which is all-cause mortality. This endpoint offers supplementary clinical data to the primary physiological endpoint, enabling an evaluation of the overall efficacy of the treatment. Moreover, the subsequent secondary endpoints have been delineated for the trial.

▪ Functional Status, Hospitalizations, and Neurocognition.
  - NYHA classification.
  - MACE (death, stroke, worsening heart failure (+1 NYHA Class), CHF hospitalization, mitral valve re-intervention).
  - Angina class.
  - Neurocognitive results.
  - Peak VO2 (assessed by a cardiopulmonary stress test).
  - The rates of patients readmitted within 30 days and the long-term readmission rates for all patients, as well as for those with cardiovascular issues and heart failure, are to be determined. Furthermore, the proportion of days spent outside of the hospital setting during the follow-up period following survival will be determined.
  - LOS for the hospital stay and discharge destination.
▪ Physiologic Measures.
  - Echocardiography assessment.
  - Quantification of MR, specifically the effective regurgitant orifice area (ERO).
  - Quantifying mitral valve area.
  - LV size, function, and geometry (including LVEF, LVEDD, LVESD, LVEDVI, LVESVI, and LV sphericity).
  - Assessment of mitral valve and subvalvular structures.
  - LA dimension.
  - RV size and function.
  - MV tethering.
  - Assessing regional wall motion for left ventricular function and viability.
  - Appropriate revascularization.
▪ Safety.
  - Incidence of serious adverse events.
  - Reoperation for MR and freedom from reoperation in general.
▪ Perioperative Measures.
  - Operative time for each procedure. For the standard surgical mitral valve operation, cardiopulmonary bypass (CPB) and cross-clamp time are required.
  - Blood loss and transfusion.
▪ Quality of Life and Economic Measures.
  - Change in quality of life (QOL).
  - Minnesota Living with Heart Failure (MLHF) score.
  - SF-12.
  - EuroQoL.
  - Dasi.

### 2.3. Therapeutic Interventions

In order to provide the most accurate and comprehensive assessment of primary and secondary endpoints, two distinct patient subgroups will be examined. The initial cohort will consist of patients who underwent TEE) or S-SMVp in conjunction with coronary revascularization, specifically PCI or CABG. The subsequent cohort will comprise patients who did not receive revascularization [14,15,16,17].

▪ Standard Mitral Valve Intervention (S-MVi) (S1 Supplementary Material).
• Mitral valve replacement (MVrpl).

MVrpl entails the preservation of the subvalvular apparatus with the objective of preventing LV diameter enlargement over time. The decision as to whether to utilize a preservation technique, prosthetic valve type, and suture positioning is at the discretion of the surgeon. In cases of ischemic cardiomyopathy, CABG is a requisite procedure for revascularization.

- Restrictive mitral annuloplasty.

An RMA can be carried out with the use of a complete rigid or semi-rigid annuloplasty ring that has been reduced in size to align with the annulus diameter. Given that patients who receive RMA frequently present with CAD, a CABG procedure may be required to facilitate optimal LV remodeling.

- Restrictive mitral annuloplasty plus subvalvular repair.

RMA may be in conjunction with the use of SV-r. Subvalvular Repair allows for the approximation or relocation of papillary muscles that are displaced by post-infarction scar formation. In individuals who have undergone SV-r due to ischemic cardiomyopathy, CABG is a necessary subsequent procedure.

▪ Transcatheter Mitral Valve Intervention (TMVi) (S2 Supplementary Material) [21,22,23,24,25,26,27,28,29,30,31,32,33,34].
• Transcatheter edge-to-edge repair (TEER).

The TEER intervention entails the convergence of the margins of the anterior and posterior leaflets of the mitral valve, a process known as edge-to-edge repair. In the event that a single MitraClip device is insufficient to achieve the desired reduction in MR, a subsequent implant may be employed to optimize the intervention.

▪ Endpoint Definitions and Measurement.
• Primary endpoint.

The primary objective of the trial is to evaluate the efficacy of the intervention in terms of left ventricular remodeling. This will be accomplished by quantifying the change in the Left Ventricular End-Diastolic Volume Index (LVESVI) at the time of hospitalization and at regular intervals following the procedural intervention, employing transthoracic echocardiography (TTE).

- Secondary endpoints.

The main secondary endpoint is mortality, which will be evaluated based on the total number of deaths from any cause.

In addition to the primary endpoints, the trial has defined the following secondary endpoints:

- Perioperative management.

The variables to be evaluated are as follows: operative time, cardiopulmonary bypass time, cross-clamp time, blood loss, and transfusions.

○ Cardiopulmonary bypass parameters.

Information pertaining to the elapsed time of myocardial ischemia, the duration of cardiopulmonary bypass, and the method of cardiac cardioplegia perfusion (retrograde or antegrade) will be extracted from the dataset.

○ Blood loss and transfusions. Supplementary Table S1 [35,36,37]
○ Reoperation for bleeding. Supplementary Table S1 [35,36,37]

A reoperation to reopen the chest for the management of excessive bleeding is indicated in cases where bleeding persists. A reoperation for bleeding is defined as any subsequent surgical procedure necessitated by the necessity to address excessive bleeding in a patient in whom the sternum was left open during the initial operation. It is essential to recognize that reopening the chest for hemodynamic instability without severe bleeding and for the purpose of puncturing the pericardium or pleura or inserting a chest tube for retained blood syndrome is not regarded as a reoperation for bleeding.

▪ Functional Status.
• MACE (major adverse cardiac event).

A MACE is specified as an unweighted composite score. It consists of the following components:

○ Death.
○ Stroke.
○ Mitral valve re-intervention.
○ Worsening heart failure (+1 NYHA Class).
○ CHF hospitalization.
• Reoperation.

All subsequent surgical procedures, including reoperations for MR, will be meticulously documented, and the incidence of reoperation will be evaluated through a rigorous time-to-event analysis.

- Peak VO2.

In contrast, functional status will be assessed by measuring maximal oxygen uptake (VO2 peak), which will be determined by conducting cardiopulmonary exercise testing at predefined intervals in patients who do not have any clinical indications that would preclude the performance of exercise testing (e.g., unstable angina or left main disease). A uniform verbal prompting protocol will be employed to encourage all patients to exercise to the point of voluntary exertion. The incorporation of Borg’s CR10 RPE (rate of perceived exertion) will be beneficial in subjects who are unable to achieve an RER (respiratory exchange ratio) of ≥1.0, providing an additional measure of the level of exertion. Accordingly, the rating of perceived exertion (RPE) will be evaluated and documented at two-minute intervals for all subjects using the RPE scale, which ranges from 0 to 10. The RPE scale will be used in all tests conducted as part of this study, and patients will be provided with laminated cards illustrating how to use the scale during these tests. A standardized protocol will be employed for the performance of cardiopulmonary exercise testing, and the results will be evaluated by the cardiologist assuming responsibility for the case at the respective enrolment center.

▪ Neurocognition.

The aim of this research is to undertake a comparative analysis of the neurocognitive profiles of the treatment groups, employing a battery of tests to assess their cognitive performance. The tests used in this study include the Hopkins Verbal Learning Test, Trail making Tests A and B, MCG Complex Figures, Boston Naming Test, Digit Span, and Digit Symbol Substitution. Neuropsychologists, whose training was conducted by experienced professionals at each recruitment site, will administer the aforementioned neurocognitive tests. The responsibility for scoring the neurocognitive tests will be borne by the neurologists at each center of enrolment.

▪ Hospitalization.
• Index hospitalization.

The length of stay for the index hospitalization will be quantified and disaggregated by days spent in the ICU. Furthermore, the discharge location will be recorded.

▪ Readmission.

The readmission rate will be calculated for the first 30 days after the procedure and for the entire follow-up time. All admissions will be classified, including those related to cardiovascular disease and HF. For a readmission to be considered heart failure-related, at least two of the following signs and conditions of acute decompensated heart failure must be present:

- Dyspnea felt related to HF.
- Administration of vasodilators, intravenous diuretics, or inotropes.
- Pulmonary capillary wedge pressure (PCWP) or LVEDP > 18 mmHg.
- On physical assessment, rales may suggest the existence of pulmonary edema or pulmonary vascular congestion. This may be seen on radiographs.

All readmissions will be categorized by the investigating physician and adjudicated by the attending cardiologist.

▪ Days Spent Alive and Outside of the Hospital.

To assess the total number of days alive and out of the hospital between treatment groups, a pooled analysis will be performed. Additionally, the proportion of days spent out of the hospital in proportion to the total number of days spent alive following the procedure will be quantitatively evaluated.

▪ Physiologic Measures.
• Echocardiographic tests.
  ○ Mitral regurgitation will be evaluated by measurement of effective regurgitant orifice area (EROA) with both proximal isovelocity surface area (PISA) and quantitative flow methods to establish its existence and degree.
  ○ The MV apparatus and valve area will undergo evaluation through the assessment of annular shape and motion, the tethering angle and tenting area, the location and distance of papillary muscle, and the calculation of the mean trans-mitral stenotic gradient utilizing mitral inflow continuous wave Doppler.
  ○ The following parameters will be employed to evaluate the LV dimension, geometry, and function: LV size, LVEF (biplane Simpson’s rule), LVEDV index using the biplane volumetric method, LV mass, LV sphericity, radial strain, and twist.
  ○ The echocardiographic evaluation will encompass the assessment of several structural parameters, including the dimensions of the left atrium, the length and surface area of the mitral valve tethering, the angle of tethering, and the position and degree of separation of the papillary muscles.
  ○ The dimensions and functionality of the right ventricle (RV) will be evaluated through the following indices: tricuspid annular plane systolic excursion (TAPSE), peak systolic velocity, diastolic E and A velocities (as determined by tissue Doppler), and fractional area change.
  ○ Doppler flow velocity measurement will be used for the purpose of gauging intracardiac pressures and hemodynamics, with a specific emphasis on pulmonary artery pressures (PAPs) and PCWP.
  ○ The regional wall motion (LVEF and viability index) will undergo evaluation at the baseline stage and again one year post-surgery. This will enable an estimation of the extent of viability at the baseline stage and an assessment of the impact of revascularization, if any, to be made.
• Suitability of revascularization.

Prior to the surgical procedure, the coronary territories are classified as either suitable or unsuitable for bypass. Following the operation, the status of the bypass is monitored. The regions that receive coronary flow should be identified in the following manner. The following coronary territories will be identified: LAD proximal, LAD distal, proximal diagonal, distal diagonal, proximal circumflex, distal circumflex, distal dominant circumflex, right posterolateral, and right posterior descending. Quality of life.

The objective of this study is to evaluate improvements in quality of life (QOL) from baseline utilizing a comprehensive array of disease-specific instruments. These include the Minnesota Living with Heart Failure (MLHF) score, the disease-specific Duke Activity Status Index (DASI), the Short Form-12 general health status index, and the EuroQol 5-D measures of health state preference, with an emphasis on both individual and societal perspectives.

The Minnesota Living with Heart Failure Questionnaire is a quantitative assessment tool designed to evaluate the impact of heart failure and its management on patients across a range of domains, including physical, psychological, and social functioning. The DASI is a multidimensional questionnaire that assesses the cardiovascular impact of four domains of adult activity, each associated with distinct levels of oxygen uptake: ambulation, personal care, domestic tasks, sexual function, and the pursuit of leisure activities. The SF-12 represents a comprehensive quality-of-life assessment tool encompassing eight distinct dimensions: physical activity, social activity, role/physical, body pain, general mental health, role/emotional, vitality, and general health perception. The SF-12 and the EuroQoL 5-D are both instruments designed to assess health-related quality of life. The EuroQol 5-D is a psychometric instrument that yields a succinct descriptive profile comprising five distinct dimensions. The instrument evaluates five domains: (i) anxiety/depression, (ii) pain/discomfort, (iii) usual activities, (iv) self-care, and (v) mobility. Furthermore, it incorporates an evaluation of the individual’s perception of their own health status.

▪ Safety.
  • Reoperation.
  • All reoperations, particularly those for mitral regurgitation, will be recorded. Freedom from reoperation will be evaluated.
  • Adverse events.

An adverse event (AE), as defined by the International Council for Harmonisation of Technical Requirements for Pharmaceuticals for Human Use (ICH), is any event that occurs during a clinical trial and that results in any of the following: a change in the condition of the study participant; a change in the participant’s status; or a new disease or syndrome that the participant develops. An adverse event must be considered clinically significant. Furthermore, it must occur in a participant who is taking part in the trial, regardless of whether the event is related to the study intervention. A pre-existing pathological state will only be designated as an adverse event if there is a discernible alteration in its character, severity, or degree.

A comparison will be made between the two groups with respect to the occurrence of any adverse effects that are deemed to be significant throughout the period of the experimental trial. The onus for adjudicating all serious and protocol-defined adverse events (AEs) will be borne by the clinical control specialists responsible for overseeing this study at each research center. Adverse events will be documented in accordance with specified safety endpoints. The report will provide detailed information on the incidence and prevalence of serious adverse effects, including the frequency of each adverse occurrence, the incidence rate per patient per annum, and the temporal distribution of adverse events. Moreover, the incidence of patients presenting with each discrete category of serious adverse event will be recorded. Safety metrics will be gathered throughout the duration of the patient follow-up period. Subsequently, the incidence of each event type will be calculated with 95% confidence intervals in alignment with the specified safety endpoints.

○ Serious adverse event.

In alignment with the directives set forth by the FDA, an adverse event is classified as serious when it results in death, is life-threatening, causes significant or prolonged disability, requires protracted hospitalization, gives rise to a birth defect or congenital abnormality, or, at the discretion of the investigative team, presents other notable hazards or potential for serious injury to the study enrollees or other individuals. In the opinion of the attending clinician, it may be considered appropriate to categorize an episode that is neither lethal nor life-threatening and does not necessitate hospitalization as a serious adverse event. This is on the grounds that a reasonable medical judgment may exist that such an episode may endanger the patient and necessitate medical or surgical intervention in order to avert one or more of the potential outcomes listed in the definition. The subsequent list provides exemplars of medical episodes that may require medical or surgical attention. Such conditions include bronchospasm necessitating intensive treatment in an emergency department or at home, blood dyscrasias, and convulsions that do not result in the need for in-patient hospitalization.

○ Unexpected major adverse events.

An unanticipated major adverse incident is defined as a critical adverse occurrence that does not fall within the parameters set forth in the established clinical trial protocol or is not duly recorded in the documentation provided to the patient for review and signature. In the event of an unexpected serious adverse event, it is mandatory to provide unscheduled notification.

In the course of the follow-up period, the occurrence of any adverse events will be duly recorded. The onus will be incumbent upon the investigating physician to ascertain whether a causal relationship exists between the adverse incident in query and the intervention that has been undertaken. The concept of causality is elucidated in Table 5.

▪ Specific Adverse Event Definitions.

A myocardial infarction (MI) is defined as a clinical syndrome characterized by the presence of at least one of the following diagnostic criteria, which collectively indicate myocardial necrosis and are consistent with myocardial ischemia [38]. These criteria include the following:

- Myocardial infarction.

The presence of elevated cardiac biomarkers (preferentially troponin) with a minimum value exceeding the 99th percentile of the upper reference limit (URL), accompanied by evidence of myocardial ischemia through the demonstration of at least one of the following:

○ New electrocardiogram (ECG) changes indicative of a new episode of ischemia, such as new ST-T changes or new left bundle branch block (LBBB), may be observed.
○ Ischemia signs.
○ An ECG examination has revealed the presence of pathological Q waves.
○ New evidence of loss of viable myocardium or new regional wall motion abnormalities should be sought.
• Peri-CABG myocardial infarction.

In subjects presenting with normal baseline troponin values prior to CABG, elevations in cardiac biomarkers above the 99th percentile URL are indicative of peri-procedural myocardial necrosis. It is customary to consider biomarker elevation exceeding five times the 99th percentile URL, in conjunction with the emergence of new Q waves or a newly diagnosed left bundle branch block (LBBB), the formation of a new graft or native coronary artery occlusion as evidenced by angiography, or imaging findings suggestive of new loss of viable myocardium, as indicative of a CABG-related myocardial infarction.

- Peri-percutaneous intervention (PCI) myocardial infarction.

For patients who have been treated with PCI and have normal baseline troponin values, any rises in cardiac biomarkers above the 99th percentile upper reference limit indicate the occurrence of peri-procedural myocardial necrosis. It has been standard practice to define PCI-related MI based on the observation of biomarker elevations greater than threefold the 99th percentile upper reference level. Furthermore, there is a recognized correlation between this subgroup and documented instances of stent thrombosis. A death that is sudden and unexpected, in the absence of any apparent cause, is classified as sudden cardiac death. This can be linked to cardiac arrest and may be followed by symptoms that indicate myocardial ischemia. In cases where this occurs, the death may be triggered by the onset of new ST elevation or new LBBB, as well as the presence of fresh thrombus, as identified by coronary angiography and/or autopsy. In the event of a death occurring before the scheduled blood sample collection or the anticipated appearance of cardiac biomarkers in the blood, the cause of death is deemed to be myocardial infarction.

- Cardiac arrhythmias.

The presence of any documented arrhythmia resulting in clinical impairment, including but not limited to hemodynamic impairment, oliguria, presyncope, or syncope, necessitates either hospitalization or a physician visit. Additionally, any arrhythmia that manifests during the course of hospitalization requires the same course of action [33,34].

○ The persistence of ventricular arrhythmia necessitates defibrillation or cardioversion.
○ The persistence of supraventricular arrhythmia necessitates the administration of pharmacotherapy or cardioversion.
• Right-sided heart insufficiency.

Persistent right ventricular dysfunction can be identified by a central venous pressure (CVP) exceeding 18 mmHg and a cardiac index below 2.0 L/min/m^2^, provided there is no evidence of elevated left atrial/pulmonary capillary wedge pressure (>18 mmHg), tamponade, ventricular arrhythmias, or pneumothorax. These findings should be considered indicative of persistent right ventricular dysfunction. In some instances, medical intervention may necessitate the implantation of a right ventricular assist device (RVAD), the administration of inhaled nitric oxide, or inotropic therapy for a period exceeding seven days [33].

- Major infection.

The emergence of a novel episode of clinical infection, characterized by the presence of pain, elevated fever, purulent drainage, and/or an increased white blood cell count, within the context of antimicrobial therapy (non-prophylactic). In cases where infection is present, a culture from the affected site or organ should yield positive results unless there is compelling clinical evidence indicating that treatment is necessary despite negative cultures. Infection may be categorized in a number of ways, as outlined below [32].

○ Localized infection.

Infection is confined to a discrete organ or region, such as mediastinitis, with no evidence of systemic involvement, as defined by the presence of sepsis. Such a determination may be made through the utilization of standard clinical methods and may be associated with evidence of a bacterial, viral, fungal, or protozoal infection or may necessitate the implementation of empirical treatment [32].

○ Endocarditis.

The subsequent findings are consistent with the diagnosis of infective endocarditis (IE), including the presence of a fever at or above 38 degrees Celsius. Furthermore, the presence of a fever at or above 37.5 °C is a further indicator. A temperature of 5 °C or above may indicate the growth of bacteria from blood cultures. Similarly, the onset of new regurgitant murmurs or HF or the occurrence of embolic events (such as focal neurological deficits, glomerulonephritis, and renal and splenic infarcts) may also be indicative of the diagnosis. In addition, the presence of septic pulmonary infarcts and the appearance of peripheral cutaneous or mucocutaneous signs (such as petechiae, conjunctival or splinter hemorrhages, Janeway lesions, Osler’s nodes, and Roth spots) are indicative of the diagnosis. In the event of an echocardiogram identifying the presence of new intracardiac vegetation, accompanied by, or otherwise unaccompanied by, additional signs and symptoms, this should be regarded as sufficient evidence to substantiate a diagnosis of endocarditis. Transesophageal echocardiography is the recommended method for diagnosing prosthetic valve endocarditis [32].

○ Sepsis.

The presence of positive blood cultures and/or hypotension is indicative of a potential systemic involvement [32].

○ Neurologic dysfunction.

The term “neurological deficit” is used to describe any new impairment of the nervous system, including both transient and permanent conditions, as well as focal and global impairments. A standard neurological examination should be conducted by a qualified medical professional, such as a neurologist, and documented through the use of appropriate diagnostic tests and the inclusion of consultation details in the patient’s medical record. The examining physician differentiates between a transient ischemic attack (TIA) and a stroke event. A TIA is defined as a transient neurological impairment with full reversibility within 24 h, absent of evidence of infarction. Conversely, a stroke is characterized as any neurological impairment lasting more than or less than 24 h, accompanied by evidence of infarction. To ascertain the extent of neurological impairment, it is essential to administer the Modified Rankin Scale and the National Institutes of Health Stroke Scale at the time of the incident and again 90 days later.

It is requisite that each neurological event be allocated to a subcategory as follows:

- A transient ischemic attack can be defined as an abrupt episode of neurological deficit that is fully reversible within a 24 h period and does not demonstrate evidence of infarction on imaging studies.
- A cerebrovascular accident, or CVA, is a medical term used to describe either an ischemic or hemorrhagic stroke. Ischemic stroke is defined as a condition with a duration of more than 24 h, while hemorrhagic stroke is characterized by the occurrence of infarction on imaging. In the event of hemorrhagic conversion following an ischemic stroke, the classification remains consistent.
- Toxic metabolic encephalopathy (TME) is a condition characterized by impaired brain function resulting from disrupted systemic metabolism or exposure to external agents. It presents with altered levels of consciousness and awareness, as well as a lack of focal neurological signs and a negative brain scan.
- Other.
- Renal events.

The assessment of renal function will be categorized in accordance with the predicted glomerular filtration rate (eGFR), which will be determined through the application of both the Chronic Kidney Disease Epidemiology Collaboration (CKD-EPI) equation [39] and the European Kidney Function Consortium (EKFC) equation [40]. The extent of renal failure will be classified into different stages, as detailed in Table 6.

Two distinct categories of kidney-related events will be identified and defined:

○ Renal dysfunction.

Abnormalities in kidney function will be characterized by a rise in serum creatinine (Cr), exceeding 100% from the established baseline, and a Cr concentration exceeding 2.0.

○ Renal failure.

A new criterion has been established for the initiation of hemodialysis in patients with renal dysfunction. It should be noted that the aforementioned definition excludes aquapheresis procedures that are solely intended for the removal of excess fluid volume.

- Hepatic dysfunction.

Hepatic dysfunction is diagnosed when any two liver tests (total bilirubin, aspartate aminotransferase/AST, and alanine aminotransferase/ALT) exceed the upper normal limit by threefold. It is also diagnosed as the primary cause of death if liver dysfunction is identified.

- Respiratory insufficiency.

The inability to discontinue ventilatory support within 48 h after surgery, which necessitates re-intubation or tracheostomy, is indicative of respiratory function impairment. This definition does not pertain to instances where the patient is re-intubated for a further surgical procedure or where the patient is temporarily intubated for diagnostic or treatment purposes.

- Bleeding.

An incident characterized by bleeding is any instance where one of the following occurs.

○ The transfusion of more than 10 units of red blood cells within the initial 24 h period following surgery has been identified as a potential risk factor for death due to hemorrhage.
○ In cases of hemorrhage or tamponade, reoperation may be required.

NOTE: A hemorrhagic stroke is categorized as a neurological incident, separate from the occurrence of bleeding.

- Pericardial fluid trapping.

The term “pericardial fluid accumulation” denotes the accumulation of either fluid or thrombus within the area of the pericardium. In instances where surgery or percutaneous catheter drainage is deemed necessary, this diagnosis must be made. The aforementioned event may be classified further into two categories: firstly, those instances that present with the clinical signs of tamponade, such as an increased central venous pressure and a decreased cardiac output, and secondly, those that do not present with these signs of tamponade.

- Arterial non-CNS thromboembolism.

A definitive assessment of an acute systemic arterial perfusion deficit in any non-cerebrovascular organ system resulting from thromboembolism can be achieved through the confirmation of one or more of the evidence-based criteria outlined below:

○ The patient was subjected to standard clinical and laboratory examinations, as well as an operative evaluation of the relevant findings.
○ Findings from the postmortem.

The provided delineation does not include occurrences pertaining to the neurological domain.

- Wound dehiscence.

The surgical incision has been compromised as a result of an event that did not involve infection. This necessitates surgical repair.

- Venous thromboembolic event.

The presence of a venous thromboembolic event, such as deep vein thrombosis or pulmonary embolism, can be identified through the utilization of established diagnostic techniques.

• Other.
▪ A critical incident is defined as a clinically significant alteration in the patient’s health state, an event resulting in death, permanent disability, or hospitalization, or an increase in the length of hospitalization. Supplementary Tables S1–S3 in Supplementary Material [41].

## 3. Statistical Methods

The presentation of continuous variables will adhere to the prescribed statistical methodology, as delineated in the relevant sections of this study. The reported variables will comprise either the mean and standard deviation or the median and interquartile range, as required. With regard to dichotomous and nominal variables, the resulting data will be presented in the form of a tally and the corresponding percentage. A range of statistical tests will be employed in the univariate analysis, including the Mann–Whitney U-test, Student’s *t*-test, Kruskal–Wallis test, Wilcoxon test, Fisher exact test, Chi-square test, and Kaplan–Meier test. In the event of incomplete datasets, no attempts will be made to supplement the information in question.

Multivariable analyses will be conducted employing the logistic regression model and the Cox proportional hazards model. The variables to be included in the logistic regression analysis will be selected using the Lasso-based variable selection method. Any noteworthy inconsistencies between the study groups will be addressed through the application of propensity score weighting. To estimate the causal effect of exposure on outcomes, a doubly robust approach will be employed comprising combination regression with inverse probability treatment weighting (IPTW) by propensity score. Multivariable analyses will be conducted by employing the logistic regression model and the Cox proportional hazards model. The variables to be included in the logistic regression analysis will be selected using the Lasso-based variable selection method. Any noteworthy inconsistencies between the study groups will be addressed through the application of propensity score weighting. To estimate the causal effect of exposure on outcomes, a doubly robust approach will be employed comprising combination regression with inverse probability treatment weighting (IPTW) by propensity score. In order to accomplish this, a propensity score that balances for covariates will be developed with the objective of minimizing discrepancies between the groups in question.

A total sample population of 630 patients was calculated as sufficient to detect a significant difference in the rate of reverse left ventricular remodeling, with 384 patients undergoing surgical mitral valve repair and 246 patients undergoing transcatheter mitral valve repair. This study was powered at 80% with a two-tailed alpha level of 0.05. In order for a *p*-value to be deemed statistically significant, it must be less than 0.05. All statistical analyses were performed with the aid of the R software package developed by the R Foundation for Statistical Computing in Vienna, Austria.

## 4. Ethics and Dissemination

### 4.1. Implications for Treating Patients Undergoing Transcatheter Versus Standard Surgical Mitral Valve Operation for Secondary Mitral Regurgitation

A thorough examination of the data from this registry will yield current insights for a substantial number of SMR patients with a comprehensive follow-up period. The multicenter nature of this registry is expected to reduce the risk of bias related to institutional volume and surgical experience. It is anticipated that all participating centers will perform a minimum of 25 procedures per annum for SMR. Furthermore, a plan for mitral surgery must be devised and implemented, allowing for effective follow-up and management of late occurrences following replacement surgery or primary repair of SMR. It is anticipated that the data collected will yield insightful information regarding the influence of distinct surgical approaches on conventional mitral valve surgery and TEER. This will facilitate the evaluation of LV remodeling in long-term follow-up. Furthermore, this research will offer conclusive data on the effectiveness of various standard mitral valve surgical techniques in a setting where mortality rates are elevated, with a 50% mortality rate, as well as long-term mortality rates for patients who have undergone TEER.

A comparison of the results obtained from the analysis of S-SMVp, which involved three distinct procedures, and TMVp for use in SMR procedures, was conducted. The following comparative analysis is based on data from a multicenter dataset, which allows for a comprehensive overview of the findings. The objective is to identify the most effective intervention for left ventricular remodeling and enhanced left ventricular ejection fraction within a 10-year timeframe. It would be beneficial to ascertain whether there is a difference in the incidence of death from all causes at 10 years following S-SMVp or TMVp. It would also be advantageous to determine whether patients with increased LVEF also have more favorable HF symptoms. A related question is which of the two procedures (S-SMVp and TMVp) achieves an immediate reduction in MR to moderate or lower levels and what proportion of individuals can derive benefit from this result.

### 4.2. The Lack of Reliable Data in Current Guidelines Was the Driving Motivation for This Study

The comparison between S-SMVp and TMVp for the treatment of SMR has been limited in observational studies [42,43,44]. S-SMVp was also compared with RMA and TEER in one subset of the Endovascular Valve Edge-to-Edge Repair Study (EVEREST) [44]. There was no significant difference between TEER and RMA in the composite endpoint of death, MV surgery or reoperation rate, and 3+ or 4+ SMR at 5 years (28.6% vs. 40.5%; *p* = 0.43) in the subgroup of patients with SMR in EVEREST (*n* = 56) [45]. Notably, these outcomes were derived from patients who had undergone a variety of surgical procedures, including both repair and replacement. For example, Kortlandt et al. [46] conducted a comparative analysis between 365 SMR patients who underwent TEER treatment and 95 patients who underwent MV surgery. Their findings revealed no statistically significant difference in adjusted survival rates up to three years post-treatment (HR, 0.86; 95% CI, 0.54–1.38; *p* = 0.541). Prior research [43,44] undertook a comparative analysis between S-SMVp utilizing RMA and TEER in a cohort of unselected SMR patients. In a retrospective analysis of 76 cases undergoing RMA and 95 cases undergoing TEER, RMA was associated with more pronounced reductions in MR, along with comparable adjusted survival rates at the six-month mark [43]. Similarly, in a distinct investigation, 65 patients received RMA treatment, while 55 were administered TEER. The results demonstrated that RMA was associated with a more pronounced and sustained reduction in MR, as well as equivalent unadjusted survival outcomes, at a median follow-up period of four years [44].

In a recent study, Okuno and colleagues [21] evaluated the efficacy of surgical repair with RMA compared to TEER for SMR, reporting the two-year outcomes. The study draws attention to discrepancies in the 2020 American Heart Association/American College of Cardiology (AHA/ACC) guidelines regarding the indication for transesophageal echocardiography (TEE) in SMR, which was classified as class IIb with level of evidence B-R.2 [3]. This investigation, which was released immediately following the publication of the new AHA/ACC guidelines, involved the evaluation of surgical versus transcatheter repair for SMR in a propensity-matched cohort of 202 patients. Following a two-year follow-up period, the authors observed no significant difference in survival rates (*p* = 0.909). However, RMA with coronary revascularization was found to be more effective than TEER in reducing MR, improving ventricular ejection fraction, and reducing New York Heart Association functional class III or IV [42]. Additionally, Okuno and colleagues discovered that RMA was superior to TEER in the long term, specifically as a secondary endpoint [42]. Evidence from the Cardiothoracic Surgical Trials Network (CTSN) and the Pulmonary Muscle Approximation (PMA) RCT trials indicated that RMA had a higher recurrence rate of MR at two and five years of follow-up, at 58.8% and 55.9%, respectively [8,11].

At present, there is a paucity of data concerning the efficacy and safety of RMA in the treatment of patients with HF and SMR. The extant studies are small observational studies that suggest improvement in left ventricular function and functional status [47,48]. Nevertheless, the inclusion criterion for RMA suitability should comprise a total of 74 patients from the CTSN trial. The group of patients presented with preoperative left ventricular end-systolic diameter and impaired apical leaflet tethering. At the two-year follow-up, patients with severe ischemic mitral regurgitation who had not experienced prolonged or recurrent mitral regurgitation following RMA exhibited significantly reduced LVESV (43 ± 26 mL/m^2^) in comparison to those who had (63 ± 27 mL/m^2^). Furthermore, the left ventricular end-systolic volume was significantly lower in this group than in patients who underwent mitral valve replacement (61 ± 39 mL/m^2^) [8].

Left ventricular remodeling has been demonstrated to be an adverse prognostic indicator in patients with ischemic heart disease. It may, however, be reversible with recovery of viable myocardium, thereby offering the potential for improved outcomes [11,12,13]. In the CTSN study, the majority of patients (75%) underwent CABG at the same time, which precluded the potential for improved regional wall motion in the remaining 25% of patients [8]. Prior research has demonstrated that the concurrent implementation of the SVR procedure and RMA is more efficacious than RMA alone, irrespective of whether the underlying pathology is ischemic or non-ischemic cardiomyopathy [11,12,13,20]. The PMA RCT comprised the randomization of 96 patients with severe chronic ischemic mitral regurgitation who had undergone complete surgical myocardial revascularization to either isolated RMA or PMA plus RMA, with follow-up for a period of 5 years. At the five-year follow-up, there was a statistically significant improvement in left ventricular end-diastolic diameter, with a mean reduction of 5.8 ± 4.1 mm and an increase of 0.2 ± 2.3 mm, respectively (*p* < 0.001). The beneficial effects observed immediately following surgery were sustained, with a significant reduction in the incidence of major adverse cardiac events (*p* = 0.004) [11,20,49,50,51].

The COAPT trial demonstrated that TEER led to a lower rate of hospitalization for heart failure and lower all-cause mortality through the 5-year follow-up in patients with heart failure and severe secondary mitral regurgitation who remained symptomatic despite the use of maximal doses of medical therapy and other indicated treatments [16]. This result was achieved despite the protocol-permitted crossover treatment of severe mitral regurgitation in patients in the control group after 2 years [17]. The TEER procedure yielded positive results across all predefined subgroups, with consistent reductions in the risks of death and hospitalization for heart failure, regardless of patient age, sex, mitral regurgitation severity, left ventricular function and volume, cause of cardiomyopathy, and surgical risk. Nevertheless, despite the favorable risk–benefit profile of TEER, adverse outcomes continued to occur in both groups. This resulted in 73.6% of the patients in the device group and 91.5% of those in the control group either dying or being hospitalized for heart failure within five years. These findings highlight the necessity for additional therapeutic options to address the underlying left ventricular dysfunction in this high-risk population. By addressing the volume and pressure overload associated with MR, TEER offers a promising solution for improving symptoms and prognosis in patients with heart failure. It should be noted that left ventricular cardiomyopathy, the underlying disease in the majority of patients with secondary mitral regurgitation, is not directly affected by MV repair. As a result, cardiovascular and non-cardiovascular events continued to occur over time, even after successful transcatheter edge-to-edge repair. This finding reflects the advanced age and multiple co-existing conditions in the trial population [17]. The level of care provided to patients with heart failure saw a notable evolution during the COAPT trial [52,53,54]. There was a marked increase in the use of angiotensin receptor–neprilysin inhibitors over the course of the follow-up period, particularly among patients in the device group. This is likely attributable to the improved hemodynamics observed following mitral transcatheter edge-to-edge repair.

The COAPT trial revealed no discernible enhancement in left ventricular remodeling with the utilization of TEER. The LVEDV was observed to be 194.4 ± 69.2 vs. 192.2 ± 76.5 mL (16, 17, 55). Nevertheless, TEER was demonstrated to facilitate superior outcomes with regard to symptom palliation and quality-of-life assessments when compared with GDMT alone. The results of a recently conducted investigation, which included the analysis of 58 patients who were initially treated with GDMT and subsequently underwent a TEER procedure, revealed a notable reduction in the composite rate of death or readmission for HF in this cohort when compared to those who were solely administered GDMT (*p* = 0.006) [16,17,55]. Furthermore, patients who underwent TEER exhibited enhanced functional status and reduced MR severity at the 3- and 5-year follow-up assessments [17,37].

Further research is required to identify the optimal threshold values for left atrium and LV size and function, which will enable the differentiation between AFMR and mixed AFMR/VFMR. It is imperative that clinical trials be conducted to compare these treatments. Furthermore, it is crucial to ascertain whether the incorporation of these strategies with the reinstatement of normal sinus rhythm results in heightened efficacy and enhanced outcomes. There is a compelling rationale for the utilization of transcatheter heart valves in the mitral position, and further studies should investigate their efficacy in treating patients with AFMR with greater assurance. Further investigation into the molecular basis of the endothelial-to-mesenchymal transition will undoubtedly facilitate the development of effective new treatments for patients who do not have sufficient growth of their valve leaflets. Additionally, a randomized trial comparing the efficacy of medication with that of early surgical intervention in patients with AFTR would be a valuable contribution to the existing literature [56].

The five 2020 AHA/ACC recommendations were all classified as level of evidence B-R or B-NR. The absence of recommendations based on reports with a 5-year follow-up indicates a moderate quality of the studies in question [3,19]. The extant literature is deficient in RCTs involving substantial cohorts of subjects, including those undergoing TEER, MV replacement, or MV repair with or without concomitant subvalvular repair surgery. The only sources that cite RCTs based on TEER with three-year outcomes are the COAPT trial [16,17,55] and the new pathophysiological framework analysis included in the American College of Cardiology/American Heart Association (ACC/AHA) guidelines [18] regarding the mechanism of SMR. At the present time, there is a dearth of compelling data to corroborate the efficacy of subvalvular repair [2,3,19].

## Figures and Tables

**Table 1:** Recommendations on the criteria for mitral valve intervention in chronic severe secondary mitral regurgitation ^a^ from international guidelines.

ESC #		
Recommendations	Class ϕ	Level ϕϕ
Valve surgery/intervention is recommended for patients with severe SMR who remain symptomatic despite GDMT (including CRT if indicated). The decision to proceed with this intervention must be made by a structured collaborative heart team.	I	B
**Patients with additional cardiac conditions, such as coronary artery disease, that necessitate therapeutic intervention**.		
It is recommended that patients undergoing CABG or other forms of cardiac surgery undergo valve surgery.	I	B
In patients presenting symptoms ^b^ that preclude surgical intervention, the heart team may determine that PCI and/or TAVR are the optimal strategies, potentially followed by TEER in cases of persisting severe MR.	IIa	C
**Patients who do not present with concomitant coronary artery or other cardiac disease necessitating intervention.**		
In patients who are not eligible for surgery and present with selected symptoms, TEER should be considered as a potential treatment option. This approach may be particularly beneficial for individuals who meet the criteria suggesting an increased chance of responding to the treatment.	IIa	B
In patients presenting with symptoms and deemed suitable for surgical intervention by the heart team, valve surgery may be recommended as a potential treatment option.	IIb	C
In patients with high-risk symptomatic conditions who are not eligible for surgical intervention and do not meet the criteria indicating a greater likelihood of responding to TEER, the heart team may, in select cases, consider a TEER procedure or other transcatheter valve therapy if applicable. This decision is made after a comprehensive evaluation of the potential benefits and risks of ventricular assist device implantation or heart transplantation. ^c^	IIb	C
**ACC/AHA** **#**		
In patients presenting with chronic severe SMR related to LVEF < 50% who continue to experience persistent severe symptoms (NYHA class II, III, or IV) despite optimal GDMT for HF) (Stage D), TEER), repair represents an effective therapeutic option in subjects with suitable anatomy, as defined on TEE, with LVEF between 20% and 50%, LVESD #70 mm, and pulmonary artery systolic pressure #70 mmHg.	IIa	BR
In cases exhibiting severe SMR (Stages C and D), MV operation constitutes a suitable intervention when CABG is employed for the treatment of myocardial ischemia.	IIa	B-NR
In cases where patients have been diagnosed with chronic severe secondary MR resulting from atrial annular dilation, whose LVEF is above 50%, and who are experiencing severe persistent symptoms (classified as NYHA III or IV) despite undergoing therapy for HF and for comorbidities such as AF, surgery on the MV may be an avenue for consideration.	IIb	B-NR
In patients presenting with chronic severe SMR, associated with LVEF < 50%, and exhibiting persistent severe symptoms (NYHA class III or IV) despite the utilization of optimal GDMT for HF Stage D, the potential merits of MV surgery should be carefully considered.	IIb	B-NR
In patients with CAD and chronic severe SMR resulting from LVEF < 50% (Stage D), MV replacement may be a reasonable option in cases where patients present with severe symptoms (NYHA class III or IV) that persist despite optimal GDMT for HF. This may be preferable to downsized annuloplasty repair, which may not provide the same level of chordal preservation.	IIb	BR

^a^ In accordance with ESC guidelines for SMR quantification, an EROA of >30 mm^2^ by 2D proximal isovolumetric surface area is likely to correspond to severe SMR. It is of the utmost importance that the quantification of SMR is conducted under optimal conditions and in accordance with the guidelines for directed medical treatment. ^b^ LVEF, predicted surgical risk, amount of myocardial viability, coronary anatomy/target vessels, type of concomitant procedure needed, TEER eligibility, likelihood of durable surgical repair, need of surgical mitral replacement, local expertise. ^c^ COAPT criteria (Cardiovascular Outcomes Assessment of the MitraClip Percutaneous Therapy for Heart Failure Patients With Functional Mitral Regurgitation). ϕ, class of recommendation; ϕϕ, level of evidence. Abbreviations: 2D, two-dimensional; AF, atrial fibrillation; CABG = coronary artery bypass grafting; CAD, coronary artery disease; CRT, cardiac resynchronization therapy; EROA, effective regurgitation orifice area; GDMT, guideline-directed medical therapy; HF, heart failure; LVEF, left ventricular ejection fraction; MV, mitral valve; HYHA, New York Heart Association; SMR, secondary mitral regurgitation; PCI, percutaneous coronary intervention; TAVR, transcatheter aortic valve replacement; TEER, transcatheter edge-to-edge repair. # Ref [2,3].

**Table 2:** Randomized clinical trial reporting secondary mitral regurgitation.

First Author or Study Acronym (Ref. *)	Type of Study	Number of Patients (N)	Treatment Option	Mean Follow-Up/Yrs	Criteria for SMR	Findings
Harmel 2019 *The Annals of Thoracic Surgery* [13]	Prospective	101	RMA (50) RMA plus PMR (51)	1	Ischemic cardiomyopathy 100%. Average patient value LVEDD > 60 mm; LVEF < 40%. EROA > 0.2 cm^2^.	Better improvement in left ventricular remodeling in PMR group. MR > 2+ more common among patients with RMA. Better survival in RMA plus PMR.
Stone 2018 COAPT *NEJM* [17]	RCT	614	TEER (302) GDTM (312)	2	Ischemic cardiomyopathy 62.5%. Average patient value LVEDV 192 mL LVEF 31 ± 9% (18% LVEF > 40%). MR Grade 3/4. EROA mean value 0.41 cm^2^; 14% EROA < 0.3 cm^2^; 41% >/= 0.4 cm^2^ 0.	Lower rate of unplanned hospitalization in TEER with disproportionate SMR. Slightly improvement in LVEDV/mL/min (from 194.4 ± 37.4 to 192.2 ± 76.5).
Iung 2019 MITRA Fr *Eur J Heart Failure* [15]	RCT	306	TEER (152) GDTM (154)	1	Ischemic cardiomyopathy 62.5%. Average patient value LVEDV 252 mL 33 ± 7% (all LVEF ≤ 40%). EROA mean value 0.31 cm^2^. 50% EROA < 0.3 cm^2^; 16% >/= 0.4 cm^2^.	No difference in unplanned hospitalization rate and death between TEER and GDTM. Slightly improvement in LVEDV/mL/min (from 136.2 ± 37.4 to 134.2 ± 37).
Nappi 2016 PMA trial *JACC* [11]	RCT	96	RMA (48) RMA plus PMA (48)	5	Ischemic cardiomyopathy 100%. Coronary artery disease with or without the need for coronary revascularization. Average value LVEDD 62 mm LVEF 42%. MR Grade 3/4. EROA > 0.2 cm^2^ or regurgitant volume > 30 mL (ESC guidelines). EROA mean value 0.34 cm^2^.	Lower rate of unplanned hospitalization in the PMA group. Better improvement in LVEDD in PMA (62.7 ± 3.4 to 56.5 ± 5.7) vs. RMA (61.4 ± 3.7 to 60.6 ± 4.6). Lower incidence of recurrent MR in the PMA group (27% vs. 55.9%)
Goldstein 2016 CTS Net *NEJM* [8]	RCT	250	MVR (125) RMA (126)	2	Ischemic cardiomyopathy 100%. Average value LVESV 63.4 mL; LVEF 40%. MR Grade 4. EROA ≥ 0.4 cm^2^ with tethering. Eligible for surgical repair and replacement of mitral valve. Coronary artery disease with or without the need for coronary revascularization.	Better improvement in LVESVI in MVR (52.6 ± 27.7 mL vs. 60.6 ± 39.0 mL). Better improvement in LVESVI in RMA with smaller LV (43 ± 26 mL/m^2^ vs. 63 ± 27 mL/m^2^). Higher incidence of recurrent MR in RMA (58.8% vs. 3.8%).

Abbreviations: EROA, effective regurgitant orifice area; LVEF, left ventricular ejection fraction; PMR, papillary muscle relocation. The other abbreviations are in the text. * Reference listed.

**Table 3:** Participating centers.

Participating Centers
Centre Cardiologique du Nord, Saint Denis, France. Department of Cardiovascular Surgery, Faculty of Medicine and Graduate School of Medicine, Hokkaido University, Sapporo, Japan. Hopital Henri Mondor, Assistance Publique—Hopitaux de Paris, Créteil, France. Biomedical Campus University, Roma, Italy. University of Genoa-UniGe, Genoa, Italy.

**Table 4:** Inclusion and exclusion criteria for patients with SMR requiring mechanical intervention.

**Inclusion Criteria**
Age 18 years or older. The patient presented with symptomatic secondary mitral regurgitation (3+ or 4+ by echocardiographic laboratory assessment), which was attributed to cardiomyopathy of either ischemic or non-ischemic etiology. The patient’s condition has been managed in accordance with the relevant clinical guidelines, including those pertaining to coronary artery disease, left ventricular dysfunction and disease, mitral regurgitation, and heart failure. Subjects with at least one hospitalization for heart failure. BNP ≥ 300 pg/mL or a corrected NT-proBNP ≥ 1500 pg/mL. NYHA functional class II or III or ambulatory class IV. Each center’s heart team will decide whether to offer MV surgery or TEER. Left ventricular end-systolic dimension ≤ 70 mm. Left ventricular ejection fraction ≥ 20% and ≤50%. The nature of the primary regurgitant jet is commissural or non-commissural. For the MitraClip group, the implanting investigator’s opinion is that the MitraClip can successfully treat the jet (if a secondary jet is present, it must be considered clinically insignificant). Trans-septal catheterization and femoral vein access is possible with the MitraClip implanting investigator. The subject gave written informed consent and agreed to all the terms of this study and the follow-up visits after the procedure.
**Exclusion Criteria**
Life expectancy is less than 12 months due to noncardiac conditions. Excessive surgical risk (in the judgment of the surgical investigator). Patients who have undergone surgery on their mitral valve leaflet in the past, have a prosthetic mitral valve implanted, or have undergone a transcatheter mitral valve procedure prior to hospitalization. The subject must have undergone transcatheter aortic valve replacement (TAVR) within 30 days prior to hospitalization. Patients who have had mitral valve surgery, prosthetic in the past, have a prosthetic mitral valve implanted, or have undergone a transcatheter mitral valve procedure before. Surgery is required for tricuspid valve disease with the exception of tricuspid valve repair. Aortic valve disease that requires surgery or transcatheter intervention. Status 1 heart transplant or previous orthotopic heart transplantation. Patients requiring emergency or urgent surgery for any reason. Preoperative Congenital heart disease (except PFO or ASD). Patients for whom transesophageal echocardiography (TEE) is contraindicated or high risk. Echocardiographic evidence of intracardiac mass, thrombus, or vegetation. Patients with active endocarditis, active rheumatic heart disease, or leaflets degenerated from rheumatic disease (i.e., noncompliant, perforated). Active infections requiring current antibiotic therapy. Patients who are pregnant or plan to become pregnant within the next 12 months. A diagnosis of chronic obstructive pulmonary disease (COPD) necessitating the administration of continuous home oxygen therapy or chronic outpatient oral steroid use. The subject must not have undergone coronary artery bypass grafting (CABG) within 30 days prior to hospitalization for a TEER procedure. Subject hospitalization is not allowed if the individual has had a cerebrovascular accident within the last 30 days. Severe symptomatic carotid stenosis, as determined by ultrasound, is greater than 70%. ACC/AHA Stage D heart failure. Chronic renal insufficiency defined by Cr ≥ 2.5 or chronic renal replacement therapy (chronic hemo- or peritoneal dialysis). In the event that the calculated pulmonary artery systolic pressure (PASP) is determined to be greater than 70 mm Hg through the utilization of echocardiography or right heart catheterization, it is regarded as a high pressure unless active vasodilator therapy within the catheterization laboratory is capable of reducing the pulmonary vascular resistance (PVR) to a level below 3 Wood Units or between 3 and 4.5 Wood Units, with a v wave less than twice the mean of the pulmonary capillary wedge pressure. Structural heart diseases that can cause heart failure, excluding dilated cardiomyopathy of either ischemic or non-ischemic etiology. These diseases include hypertrophic cardiomyopathy, restrictive cardiomyopathy, and constrictive pericarditis. Infiltrative cardiomyopathies such as amyloidosis, hemochromatosis, and sarcoidosis. The patient is experiencing hemodynamic instability and requires inotropic support or mechanical heart assistance. The echocardiogram shows moderate or severe right ventricular dysfunction, indicating physical evidence of right-sided congestive heart failure. Implant of any Cardiac Resynchronization Therapy (CRT) or Cardiac Resynchronization Therapy with a cardioverter–defibrillator (CRT-D) within the last 30 days prior hospitalization. The mitral valve orifice area should be assessed by a transthoracic echocardiogram (TTE) within 90 days prior to subject hospitalization and should be less than 4.0 cm^2^. Modified Rankin Scale ≥ 4 disability.

**Table 5:** Causality of adverse events observed during the follow-up period.

**Probable**
The following attributes will be applicable to the adverse events in question that are deemed, following a meticulous medical assessment, to be linked to the surgical procedure (S-MVi or TMVi) with a high degree of reliability. There is a clear temporal relationship between the event and the surgical procedure. The event is a representative example of the typical postoperative course, which can be ascribed to a commonly observed underlying condition among the general population or individual. The incident in question is not a documented outcome of the surgical intervention in question, and there is no reasonable basis for attributing it to an alternative cause
**Possible**
In the event that an adverse event, following a thorough clinical evaluation, does not align with the parameters of a probable relationship with the surgery, but a definitive conclusion has not been reached, it is essential to consider the following characteristics. The event in question occurs subsequent to the surgical procedure. It is not possible to attribute the adverse event to the surgery, nor can it be explained by the known, medically related conditions that have been previously observed.
**Unlikely**
The following characteristics will be taken into account in the event that, subsequent to a thorough medical evaluation, an adverse event does not meet the criteria for a potential or likely correlation with the surgery in question and a causal link does not appear to be plausible. The chronological progression of events does not align with the sequence of actions undertaken during the surgical procedure. It is not possible to discern a clear pattern of response to the surgical procedure, and it is plausible that the event may have been caused by environmental factors.

**Table 6:** Outline of the criteria for defining postoperative acute kidney injury †. † The serum creatinine level undergoes fluctuations during the course of hospitalization.

Criteria Used to Define Postoperative Acute Kidney Injury †	
Level	Creatinine in Serum
1	An increase of 1.5–1.9 times the baseline, or an increase of at least 0.3 mg/dL (26.5 micromol/L), is to be expected.
2	2 to 2.9 times the baseline.
3	An increase to 3.0 times the baseline is indicated or, alternatively, a value of 4 mg/dL (353.6 micromol/L) should be reached, at which point renal replacement therapy should be initiated.

## Supplementary Materials

The following supporting information can be downloaded at: https://www.mdpi.com/article/10.3390/jcm13247742/s1, Figure S1: Surgical technique; Table S1: Classification for bleeding during E-CABG surgery; Table S2: New York Heart Association (NYHA) Classification; Table S3: Canadian Cardiovascular Society Classification.

## Acronyms

AE	adverse event
CABG	coronary artery bypass surgery
CHF	chronic heart failure
COAPT	Cardiovascular Outcomes Assessment of the MitraClip Percutaneous Therapy
GDMT	guideline-directed medical therapy
HF	heart failure
ICU	intensive care unit
LV	left ventricle
LVEDP	left ventricular end-diastolic pressure
LVEF	left ventricular ejection fraction
MACE	major adverse cardiac events
MCG	Medical College of Georgia
MI	myocardial infarction
MV	mitral valve
NYHA	New York Heart Association
PCI	percutaneous coronary intervention
PCWP	pulmonary capillary wedge pressure
RCT	randomized clinical trials
RER	respiratory exchange ratio
RMA	restrictive mitral annuloplasty
RPE	rate of perceived exertion
SV-r	subvalvular repair
SMR	secondary mitral regurgitation
S-MVi	standard mitral valve intervention
TEER	transcatheter edge-to-edge mitral valve repair (TEER)
TEERMISO	Transcatheter Versus Standard Surgical Mitral Valve Operation for Secondary Mitral Regurgitation
TMVi	transcatheter mitral valve intervention
